# Glycomacropeptide Bioactivity and Health: A Review Highlighting Action Mechanisms and Signaling Pathways

**DOI:** 10.3390/nu11030598

**Published:** 2019-03-12

**Authors:** Laura Elena Córdova-Dávalos, Mariela Jiménez, Eva Salinas

**Affiliations:** Department of Microbiology, Basic Science Center, Autonomous University of Aguascalientes, Aguascalientes 20131, Mexico; lcdavalos@gmail.com (L.E.C.-D.); mayojv@hotmail.com (M.J.)

**Keywords:** glycomacropeptide, milk-derived bioactive peptide, antibacterial, prebiotic, remineralizing, metabolism, anti-tumoral, immuno-modulatory, action mechanisms

## Abstract

Food-derived bioactive peptides are reported as beneficial and safe for human health. Glycomacropeptide (GMP) is a milk-protein-derived peptide that, in addition to its nutritional value, retains many biological properties and has therapeutic effects in several inflammatory disorders. GMP was shown under in vitro and in vivo conditions to exert a number of activities that regulate the physiology of important body systems, namely the gastrointestinal, endocrine, and immune systems. This review represents a comprehensive compilation summarizing the current knowledge and updated information on the major biological properties associated with GMP. GMP bioactivity is addressed with special attention on mechanisms of action, signaling pathways involved, and structural characteristics implicated. In addition, the results of various studies dealing with the effects of GMP on models of inflammatory diseases are reviewed and discussed.

## 1. Introduction

Recently, scientific evidences showed that biologically active proteins and peptides derived from food could have beneficial activities on human health [1]. Food-derived bioactive peptides are inactive within the sequence of their parent protein, and can be released during gastrointestinal digestion or food processing via the action of proteolytic enzymes. Once released, they may interact with selected receptors and regulate physiological functions in the body [1]. One important source of food-borne bioactive peptides is milk. In particular, milk-derived bioactive peptides exert several important health-promoting activities, such as anti-hypertensive, anti-microbial, anti-oxidative, immuno-modulatory, and opioid- and mineral-binding properties [2]. Glycomacropeptide (GMP), also called caseinomacropeptide, is a milk-derived bioactive peptide that is released from κ-casein via enzymatic digestion, either physiologically or in industry during the cheese-making process [3]. The most studied and best characterized GMP is that of bovine origin, due to its availability in large quantities for marketing purposes. The objective of this review was to summarize literature reports on the health-beneficial biological activities of bovine GMP, including antibacterial, prebiotic, remineralizing, inhibitory of gastric secretion, modulating of gastric hormone secretion and metabolism, anti-tumoral, and immuno-modulatory activities, with a focus on mechanism actions and implicated cellular signaling. Firstly, GMP’s basic chemical structure is reviewed to understand the importance and implication of each part of the molecule in its biological properties.

## 2. Structure and Composition of GMP

**Figure 1:**
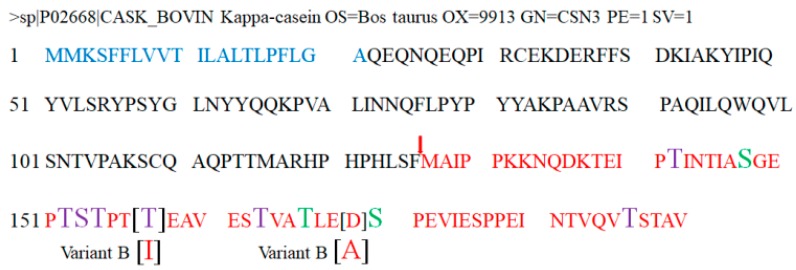
Amino-acid sequence of *Bos taurus* κ-casein protein. The sequence is reported in database UniProtKB accession number P02668. The blue-colored sequence corresponds to a signal peptide and red sequence corresponds to glycomacropeptide (GMP). Chymosine’s cleave site is indicated with red arrow. Purple amino acids indicate glycosylation sites, and green amino acids show phosphorylation sites. The modification in variant B of GMP is indicated in square brackets.

The bioactivity of peptides is due to their structural features, which allow their interaction with biological ligands and thereby the induction of their effects. Through several studies, it was possible to know structural and molecular data of GMP. GMP is produced by hydrolyzing the peptide bond of the amino acids phenylalanine-105 and methionine-106 of κ-casein via the action of chymosin during cheese making [4,5,6] or pepsin during the digestion process [7]. The complete protein sequence of κ-casein was reported for the first time by Mercier et al. [8] and can currently be obtained from the UniProt database with accession number P02668, being encoded by the *csn3* gene of *Bos taurus*. A signal peptide of 21 amino acids is located at the amino-terminus, which is proteolyzed during extracellular secretion [6,8]. The peptide fraction of GMP corresponds to the 64 carboxy-terminal amino acids of κ-casein (methionine-106 to valine-169) (Figure 1). It is important to mention that GMP does not present aromatic amino acids (phenylalanine, tryptophan, and tyrosine) or cysteine. For this reason, its use as a source of protein in phenylketonuria (PKU) patients was proposed [9], although further studies are necessary to prove its efficacy [10]. Due to the absence of aromatic amino acids, it is only detected at a wavelength of 205 to 217 nm [11,12]. Furthermore, GMP consists of only one residue of methionine and is rich in branched-chain amino acids (leucine, isoleucine, valine) [3,8].

The non-glycosylated GMP monomers have a molecular mass of 6755.47 Da for variant A and 6787.43 Da for variant B. The average molecular mass of glycosylated GMP is about 7500 Da, although the highly glycosylated form reaches 9631 Da [13]. An isoelectric point of 3.15 was reported for glycosylated GMP and 4.15 for non-glycosylated form [14]. GMP has the ability to associate in trimers (depending on the pH of the solution) and has a high solubility in water, in addition to its heat stability [3]. Theoretically, it is predicted that the GMP probably presents structures of the random coil type, and its three-dimensional configuration could be different depending on the ionic strength, pH, and presence of other molecules; the peptide has a negative partial charge except in the amino terminus, where three amino acids are found with a positive partial charge; at a pH of 7.0, the peptide its highly hydrophilic [14].

Approximately 11 genetic variants of κ-casein are known, of which two predominate in bovine milk, variants A and B; GMP variant B differs from variant A due to the substitution of a residue of isoleucine by a residue of threonine at position 136, and the substitution of a residue of alanine by an aspartic acid residue at position 148 (Figure 1) [6,8].

At the post-translational level, GMP has two types of modifications which influence the peptide properties: phosphorylation and glycosylation. Phosphorylations were reported at serine-148, threonine-166, and serine-170 of κ-casein [15,16]. In the case of serine-170, as later mentioned, this residue can also be glycosylated, and, even in the same κ-casein sample, it was detected in phosphorylated and glycosylated states [16]. The biological reasons for the alternation of post-translational modifications at serine-170 are not known yet. In relation to glycosylation states of GMP, despite decades of studies and the use of various combinations of techniques and analysis, a very high level of heterogeneity was observed in the samples due to different origins of the milk and used techniques. A common finding in all reported studies is that only *O*-glycosylations are present in serine or threonine residues [15,16,17]. Sugars were detected at threonine-142, -152, -154, -157, and -163, and at serine-153 and -170, while some authors also reported detection at threonine-186 [15,16,17]. To increase the precision of the results, other proteases could be used to digest the GMP, and the peptides obtained can be analyzed by collision-induced dissociation coupled with tandem mass spectrometry or electron-transfer dissociation mass spectrometry [16].

**Figure 2:**
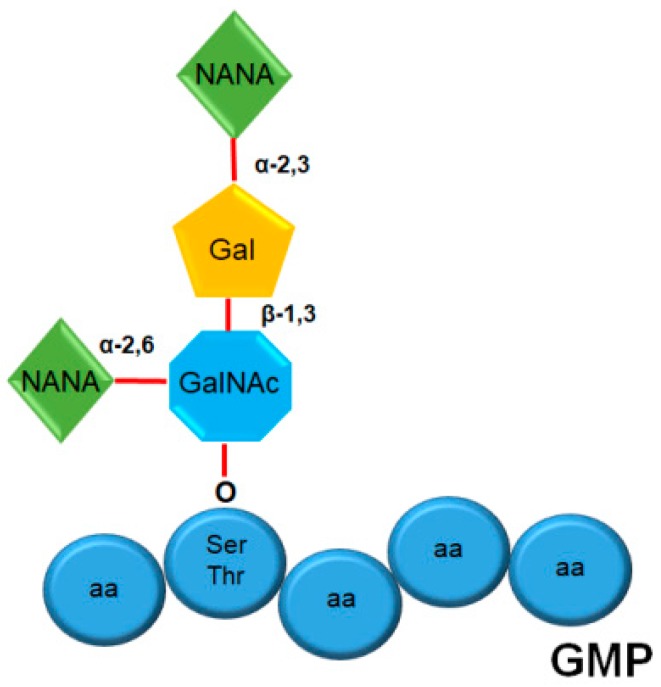
Structure of *O*-linked tetrasaccharides of glycomacropeptide. The image depicts the most abundant oligosaccharide structure in glycomacropeptide (GMP), where *N*-acetylgalactosamine (GalNAc) is *O*-linked to serine (Ser) or threonine (Thr), which is respectively bonded via α-2,6 and β-1,3 bonds to *N*-acetylneuraminyl or sialic acid (NANA) and galactosyl (Gal); in turn, Gal is linked via an α-2,3 bond to NANA. aa, amino acid.

The analysis of GMP sugar content via chemical methods showed that the GMP presents in its structure the following types of sugars: *N*-acetylneuraminyl or sialic acid (NANA), galactosyl (Gal), and *N*-acetylgalactosamine (GalNAc) [4,18]. The determination of the structure of the oligosaccharide and the type of bond between sugar molecules was carried out using fast atom bombardment mass spectrometry, and ^13^C nuclear magnetic resonance, which allowed observing that there are heterogeneous mixtures of GMP with different glycosylation units: GMP with monosaccharides, disaccharides, trisaccharides, and tetrasaccharides. In all the analyzed samples, it was observed that the sugar linked to the OH• radical of the amino acid is always GalNAc. Performing a calculation of molar proportionality of each fraction obtained by high-performance liquid chromatography (HPLC), it was determined that a tetrasaccharide linked to GMP is the most abundant form, and it has the following structure (Figure 2): GalNAc *O*-linked to serine or threonine, which is linked via α-2,6 and β-1,3 bonds to NANA and Gal, respectively; in turn, Gal is bonded via an α-2,3 bond to NANA [18].

## 3. GMP Biological Activities

**Figure 3:**
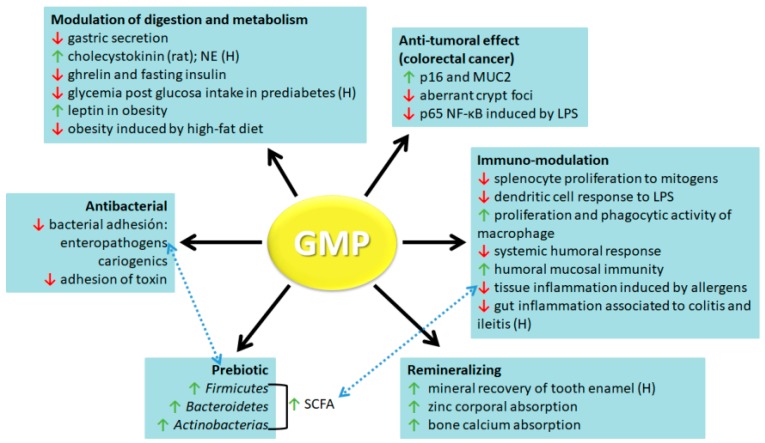
Biological activities ascribed to glycomacropeptide. The main biological activities of glycomacropeptide (GMP) with health-beneficial properties are antibacterial, prebiotic, remineralizing, modulation of digestion and metabolism, anti-tumoral, and immuno-modulation activities. Those activities demonstrated in clinical trials are indicated by (H). Red arrows indicate downregulatory effects and green arrows indicate upregulatory effects. Blue discontinuous arrows indicate associated mechanisms. NE, no effect; p16, cyclin dependent kinase inhibitor; MUC2, mucin 2; p65 NF-κB, p65 nuclear factor-κB subunit; LPS, lipopolysaccharide; SCFA, short-chain fatty acids.

As the knowledge on structural characteristics and physicochemical properties of GMP advances, there is a growing interest in studying its usefulness in the food industry. GMP showed properties as a nutritional supplement, food additive, or as a functional food. In recent decades, different researchers devoted themselves to the study of GMP biological activities. Best described ones with a positive impact on human health are antibacterial, prebiotic, remineralizing, modulatory of digestion process and metabolism, anti-tumoral, and immune-modulatory activities (Figure 3). In this paper, we address a review of the results obtained both in vitro and in vivo that show that GMP can be used as a valuable component for the development of nutraceuticals or functional foods.

### 3.1. Antibacterial

The first report about this activity was the ability of GMP to inhibit morphological changes induced by cholera toxin (CT) or *Escherichia coli* enterotoxins (LT-I and LT-II) on Chinese hamster ovary (CHO)-K1 cells, probably due to GMP inhibiting the binding of these toxins to their specific receptors, an effect only demonstrated for the CT-receptor ganglioside GM1 [19]. Later, it was reported that the inhibitory effect on damage induced by CT on CHO-K1 cells was independent of the dose, reached at least an inhibition level of 70%, and was dependent both on peptide sequence and sialic acid presence [20]. Subsequently, another study showed the dose-dependent binding ability of GMP to pathogenic bacteria, such as entherohemorragic *E. coli* (EHEC O157), *Salmonella enteritidis*, and *Morganella morganii*, but not to the probiotic bacteria *Lactobacillus casei*. The binding ability of GMP to EHEC O157 was abolished by desialylation and peroxidation, while its attachment to *S. enteritidis* was completely eliminated by peroxidation, but only reduced by asialo-GMP, suggesting that, depending on the bacteria, GMP binding is mediated by sialic acid or by other different carbohydrate molecules [21]. In this work, authors demonstrated for the first time that GMP is able to reduce the adhesion of bacteria to their target cells, particularly of EHEC O157 to Caco-2 cells. Later studies expanded the information about the bacteria whose adhesion is inhibited by GMP, including both pathogenic and probiotic ones. Among pathogenic bacteria, the inhibitory effect was demonstrated for the adhesion of verotoxigenic and entherophatogenic *E. coli* (EPEC) to HT-29 human colon adenocarcinoma epithelial cells [22]; EPEC, *S. typhimurium*, or *Shigella flexneri* to Caco-2 cells [23]; and entherotoxigenic *E. coli* (ETEC) K88 to piglet ileal mucosa cells [24]. In some cases, this activity was improved when GMP was protease-digested [23], and it was shown that GMP interacts with the bacteria, but not with their target cells [25]. In relation to probiotic bacteria, GMP reduced the binding of *L. pentosus*, *L. acidophilus*, and *L. casei* to HT-29 cells [22].

On the other hand, there are in vivo assays that consolidate the protective role of GMP against pathogenic bacteria. A dose-dependent protective effect of GMP against diarrhea induced by CT and enterotoxins LT1 and LT2 of *E. coli* was described in mice [19]. Moreover, the inclusion of GMP in the diet of weaning piglets challenged with ETEC K88 reduced the bacterial count in mucosal scrapping and the adhesion ability of ETEC to the intestine, prevented ileum colonization by enterobacteria, relieved gut morphological damage associated to infection, and protected from the alteration of intestinal barrier function [24,26].

Although there is enough evidence, both in vitro and in vivo, about the protective effect of GMP on enteropathogenic bacterial infection, more studies are needed to facilitate the understanding of the mechanisms via which it exerts its effects and to discard that GMP may induce dysbiosis. Also, it will be interesting to know whether this glycopeptide also exerts antimicrobial effects on bacteria which infect other tissues, such as airways, the skin, or the genito-urinary tract. In this sense, different studies showed an anti-cariogenic activity of GMP. The addition of GMP as an active component to different products showed that it inhibits the adhesion to surface plastic of bacteria that induce dental plaque and caries, such as *Streptococcus mutans*, *S. sanguis*, and *Actinomyces viscosus* [27]. Likewise, the incorporation of GMP into salivary films modified the adherence of *S. sobrinus* and *S. mutans* to bovine enamel discs [28].

### 3.2. Prebiotic

Studies about GMP as a prebiotic were developed by analyzing the growth of particular strains of probiotic in GMP-enriched medium and changes in gut microbiota of GMP-administered animals or humans. All studies point out the growth-promoting activity of GMP on particular microbial species, but are controversial about which part of the molecule, peptide or carbohydrate, is involved. The prebiotic activity of GMP was demonstrated for the first time on a strain of *Bifidobacteria infantis*, activity that was lost by digesting the peptide with proteases, evidencing the importance of the amino-acid sequence as a promoter of bifidobacterial growth. In addition, this activity was greater using human than using bovine GMP [29]. Later, in a new attempt to evaluate the effect of bovine GMP on *B. infantis*, *B. breve*, and *B. bifidum* growth, it was shown that the prebiotic activity was inversely proportional to GMP concentration, which was interpreted by authors as sialic acid not being very important in this activity due to its content being extremely low at low GMP concentration. However, in the same work, the effect of other sialylated proteins was also analyzed, making it evident that, as the content of sugars increases, the probiotic effect greatens [30]. With this background, it was demonstrated that the supplementation of dairy products or culture medium with GMP increases the growth of some probiotics [31,32]. A subsequent experiment developed in a model of artificial colon showed that supplementation with bovine GMP increases the growth of *Coprococcus* and *Clostridium* cluster XIVb in samples of human feces donated by healthy or frail elderly individuals, whereas genus *Dorea* was only found in high amounts in the first ones; these three microorganisms are related in the resistance to pathobiont colonization. In addition, this work was the first to prove that GMP increases the production of total short-chain fatty acids (SCFA) [33]. A recent research showed that GMP promotes the growth of *B. longum* ssp. *infantis* in a dose-dependent manner, and the effect was lost when oligosaccharides of GMP were oxidized by periodate, suggesting one more time that bacterial growing is due to the sugar molecules present in GMP [34].

Although the findings about GMP as a growth promoter of some probiotics in vitro are promising, in vivo studies are indispensable. In this way, it was found that GMP enhances the establishment of a healthy intestinal microbiota and prevents pathogenic bacteria colonization in mice. After 15 days of daily treatment with GMP and using fluorescence in situ hybridization, a significant increment in the number of *Lactobacillus* and *Bifidobacteria* was observed, together with a significant decrease in *Enterobacteriaceae* and coliforms on fecal samples of BALB/c mice [35]. Authors associated the reduction of intestinal pathogenic bacteria with the ability of GMP through its sialic acid residues to bind them, a feature previously mentioned in this review. Likewise, healthy and PKU mice receiving a GMP-enriched diet for eight weeks modified the intestinal bacterial population by reducing *Proteobacteria* phylum, especially *Desulfovibrio* sp. both in cecal content and feces, microorganisms associated with the pathogenesis of inflammatory bowel disease (IBD). On the other hand, healthy mice increased *Firmicutes* in cecal content, particularly *Allobaculum*, and PKU mice augmented *Bacteroidetes* in feces, mainly *Bacteroides*. For the first time, oral administration of GMP was associated with an increase in cecal content SCFA concentration (acetate, butyrate, and propionate) [36]. Although all these studies demonstrated the prebiotic activity of GMP, more research is necessary to clarify which fraction of the molecule is participating.

### 3.3. Remineralizing

The remineralizing effect of GMP was described for the first time in teeth and in association with the antimicrobial activity previously described. A protocol carried out in humans showed the mineral recovery of temporarily implanted demineralized bovine enamel, due to the application of toothpaste supplemented with GMP, alone or in combination with xylitol [37]. Later, and unconnected to the antimicrobial activity, it was observed that infant rhesus monkeys fed with a milk formula supplemented with GMP for four months improved zinc corporal absorption [38]. Furthermore, the feeding of male and female PKU mice with a GMP-supplemented diet for 20 weeks attenuated, regardless of sex, the deleterious effect of the disease on bones [39]. In other work, it was demonstrated that oral administration of GMP for three or eight weeks to male mice fed with a low-calcium diet improved the calcium concentration in the femur at both tested times, mainly during the period of full body development [40]. Likewise, the feeding of female mice for 29 weeks with a high- or low-fat diet supplemented with GMP as a source of proteins enhanced the bone quality [41]. Authors suggested that all these effects could be mediated by the prebiotic activity of GMP, which is associated with an increment of cecal SCFA (before being reviewed), although studies are needed to prove it.

### 3.4. Modulator of Digestion and Metabolism

Another effect attributed to GMP is the inhibition of gastric secretion. Firstly, it was reported that GMP suppresses gastric secretion triggered by gastrin and other stimulants such as histamine, and stomach motility in dogs [42,43,44]. Later, it was suggested that the inhibition of gastric secretion was caused by a low-molecular fraction of GMP, not by the whole molecule [45,46]. Thus, these studies suggest that, during natural breast feeding of newborn animals, GMP may develop an important role in the conservation of active milk proteins.

The gastric secretion inhibitory effect reported for GMP led to a study on its role in the secretion of specific gastric hormones. In this sense, GMP was associated with appetite control by modulating the secretion of cholecystokinin (CCK). In situ assays to measure stimulated CCK secretion were carried out in rats applying GMP ex vivo [47] or after administering GMP in vivo [48], showing that it is able to stimulate CCK secretion. Specifically, this effect was detonated by the glycosylated variant A of GMP [47]. However, in studies in male and female humans, GMP had no effect on the modulation of food intake over a short-term period or on satiety [49,50], or on the loss of body weight after a long-term period of sustained consumption [51]. Similarly, the intake of a preload containing GMP with different degrees of glycosylation did not change the levels of CCK in human plasma of normal and overweight subjects [50,52]. In general, authors agreed that the discrepancy between the results in murine models and human studies was due to used GMP doses [49,50]; thus, it is pertinent to continue making studies that allow defining the optimal dose that could modulate CCK secretion. In addition, trials in PKU subjects or in mice fed with high- and low-fat diets supplemented with GMP provide evidence that GMP consumption suppresses the concentration of plasmatic ghrelin, an appetite-stimulating hormone [41,53].

On the other hand, this glycopeptide is not only associated with the regulation of hormones related to food intake, but can also modulate other digestive and metabolic hormones. Thus, it was reported that the long-term ingestion of GMP-enriched whey decreased the fasting plasma insulin levels in rat, while the whey alone did not modify it [54]. Furthermore, when GMP was administered to prediabetic patients in combination with an intake of glucose, the triggered glycemia was diminished as compared with that induced by the glucose alone [55].

Additionally, an anti-obesity effect of GMP was suggested using a model of obese rats induced by a high-fat diet. Based on a previous work that showed that GMP suppresses in vitro proliferation, differentiation, and lipid accumulation of primary rat preadipocytes [56], Xu et al. [57] designed a study to analyze the effects of a high-fat diet supplemented or not with GMP on adipose tissue metabolism. It was shown that GMP reduced the body weight gain and fat accumulation in liver, epididymal, and perirenal adipocytes of obese rats. It also decreased total and low-density lipoprotein (LDL) cholesterol in plasma, cholesterol and triglycerides in liver, and liver oxidative damage, showing that GMP can reverse the detrimental effect caused by a high-fat diet. At the same time, levels of tumor necrosis factor alpha (TNF-α) and interleukin (IL)-6 in plasma of obese rats were reduced by GMP supplementation, and that of leptin was increased. Due to obesity being an important worldwide problem of public health and preventive medicine, these results propose GMP as a valuable compound in the prevention of obesity-related dysfunctions.

### 3.5. Anti-Tumoral

Recent studies suggested a protective role of GMP against cancer. In a rat model of dimethylhydrazyne-induced colorectal cancer, oral administration of GMP increased the expression of p16 and mucin2 (MUC2), proteins associated with cancer protection, while it attenuated the number of aberrant crypt foci, a cancer establishment indicator [58]. Moreover, GMP inhibited lipopolysaccharide (LPS)-induced expression of p65 nuclear factor (NF)-κB subunit in human colorectal adenocarcinoma HT-29 cells, an important mechanism of colon cancer triggering in IBD [59].

### 3.6. Immuno-Modulation

GMP was demonstrated to modulate the immune response in different manners. There are multiple in vitro studies that show a regulatory activity of GMP on different functions carried out by immune cells. Additionally, orally administered GMP is able to modulate the immune response that underlie several inflammatory pathologies. Currently available information indicates that the regulation of the activity of involved cells may be a crucial mechanism through which GMP exerts its immuno-modulatory activity in inflammatory diseases, although it is not the only one.

**Figure 4:**
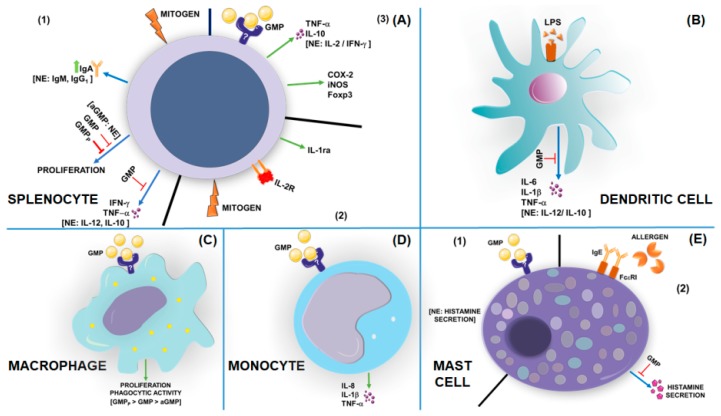
Effect of glycomacropeptide on immune cell function determined from in vitro assays. Glycomacropeptide (GMP) regulates the response of splenocytes, dendritic cells, macrophages, monocytes, and mast cells in different manners. So far, the receptor to GMP is unknown. (**A**) Splenocytes: (**1**) GMP modifies rodent splenocyte response to mitogens by inhibiting proliferative response and tumor necrosis factor (TNF)-α and interferon (IFN)-γ production, and by increasing immunoglobulin (Ig) A secretion. The inhibition on cellular proliferation is greater with proteolyzed GMP (GMP_P_) and is lost with asialylated GMP (aGMP). GMP has no effect on interleukin (IL)-12, IL-10, IgM, and IgG1 mitogen-stimulated release; (**2**) GMP stimulates the synthesis of soluble IL-1 receptor antagonist (IL-1ra) by splenocytes and suppresses the expression of the IL-2 receptor (IL-2R) on its cell membrane, when stimulated by mitogens; (**3**) in response to GMP, splenocytes secrete TNF-α and IL-10, and increase the expression of cyclooxygenase-2 (COX-2), inducible nitric oxide synthase (iNOS), and forkhead box P3 (Foxp3). GMP does not induce IL-2 and IFN-γ secretion. (**B**) Dendritic cells: GMP decreases IL-6, IL-1β, and TNF-α secretion induced by lipopolysaccharide (LPS) in murine bone-marrow-derived dendritic cells, without modifying IL-12 and IL-10 release. (**C**) Macrophages: GMP improves proliferation and phagocytic activity of human macrophages U937 when cultured at low calf fetal serum concentration. The effect is greater with GMP_P_ and less with aGMP. (**D**) Monocytes: GMP triggers IL-8, IL-1β, and TNF-α release from THP-1 monocyte cells and human primary blood monocytes. (**E**) Mast cells: GMP does not stimulate histamine release from rat peritoneal mast cells, but inhibits its release when cells are activated by allergens through the IgE–high-affinity IgE receptor (FcεRI) complex. NE, no effect.

The first studies about the immuno-regulatory activity of GMP focused on analyzing the effect on splenocyte proliferative response to mitogens, and most of them were carried out in vitro (Figure 4A). In 1992, Otani et al. [60] demonstrated that κ-casein inhibits the proliferation of mouse splenocytes induced by *S. typhimurium* LPS, with the GMP fraction responsible for this effect. In addition, GMP was reported to inhibit the proliferative response induced by concanavalin A (ConA) and phytohemagglutinin (PHA) on splenocytes [60,61]. Otani and Monnai [62] observed that the inhibitory activity of GMP on mitogen-induced proliferation of spleen cells was lost after digestion with neuraminidase, indicating that sialic acid is critical for this phenomenon. However, after GMP digestion with trypsin and pronase, its inhibitory effect was increased, which pointed out that the peptide chain is also involved (Figure 4A1). In this sense, a study in which GMP was separated into distinct fractions containing different numbers of sialic acid groups demonstrated that the inhibitory effect on PHA-induced proliferative responses improved as the numbers of sialic acid residues increased, whereas that on LPS-induced proliferation was highest with the GMP fraction containing two sialic acid residues [63]. Both inhibitory effects decreased significantly after neuraminidase digestion. Altogether, these findings indicate that both sialic acid residues and the polypeptide portion of GMP are essential for inhibitory effects on LPS- and PHA-induced splenocyte proliferation. Once it was found that GMP inhibits splenocyte proliferation induced by mitogens [64], studies focused on discovering the associated mechanism. Accordingly, it was demonstrated that GMP stimulates the synthesis of a soluble inhibitory component, an IL-1 receptor antagonist (IL-1ra) [65,66]. Also, it was reported that GMP binds to mouse cluster of differentiation 4 (CD4)-positive helper T cells and suppresses the expression of the IL-2 receptor on the cell membrane, inhibiting the PHA-induced proliferation of mouse splenocytes (Figure 4A2) [67]. However, some controversy about the effect of GMP on the in vitro proliferation of spleen cells was later generated, as Requena et al. [68] showed that GMP increases the proliferation response of lymphocytes stimulated by Con A. In relation to in vivo studies that analyze a possible immuno-modulatory activity of GMP in relation to splenocyte response to mitogens, the first one was developed in 1998 by Monnai et al. [69]. They demonstrated that mice fed with a diet supplemented with GMP enhanced the proliferative response of spleen cells to ConA, without generating significant changes in the response to LPS or PHA. However, recently, our working group showed that oral administration of GMP to rats reduced the proliferative response of splenocytes induced by ConA [70]. In summary, most studies pointed out the inhibitory effect of GMP on splenocyte proliferation to mitogens. The different responses reported by Monnai [69] and Requena [68] groups were probably due to concentration-dependent effects or assay-used conditions.

Apart from splenocytes, other immune cells were analyzed to understand the immuno-modulatory properties of GMP. In this sense, it is known that GMP induces a slight but significant decrease in the production of IL-6, IL-1β, and TNF-α by murine bone-marrow-derived dendritic cells in response to LPS, without affecting that of IL-12 and IL-10 (Figure 4B) [67]. GMP also improves proliferation and phagocytic activity of the human macrophage-like cells U937 generated at low calf fetal serum concentration, with this effect being greater when GMP was pepsin-digested or a sialic acid-rich fraction was used, as compared with the effect induced by non-digested or asialo GMP (Figure 4C) [71]. When used in combination with other substances, GMP potentiates the action of α-lactalbumin and β-lactoglobulin on the stimulation of chemotaxis, superoxide production, and degranulation and phagocytosis of human neutrophils [72].

There are several studies that analyzed GMP effects on humoral immunity. Mice fed with GMP showed suppressed levels of specific immunoglobulin (Ig)G to dietary and injected antigens, with no change in IgM, IgA, and IgE antibody response [69]. In addition, Yun et al. [73] studied the effect of GMP on the production of Igs by splenocytes stimulated with LPS, finding that only IgA levels were increased by GMP, as well as the population of IgA-positive cells (Figure 4A1). In this sense, a recent study demonstrated that oral GMP administration to mice resulted in a greater number of IgA-positive plasma cells in the intestinal lamina propria [74]. Accordingly, other work showed that GMP intake recovers the levels of secretory IgA in intestinal mucosa of mice with experimental ulcerative colitis [75]. Altogether, these results suggest an immuno-suppressing activity of GMP on systemic humoral response, but an immuno-stimulating activity on humoral mucosal immunity.

A Spanish group led by Martínez-Augustin studied the immuno-modulatory action of GMP in experimental models of intestinal inflammation (revised in detail in Table 1). They demonstrated that orally administered GMP to rats exerts an anti-inflammatory effect in colitis and ileitis induced with trinitrobenzenesulfonic acid (TNBS), or in colitis induced by dextran sulfate sodium (DSS), with a degree of efficacy in some assays similar to that of sulfasalazine, a drug widely used in the therapy of IBD (Table 1) [76,77,78]. In the TNBS-induced colitis model, GMP was probed as pre- and post-treatment, and it offered superior anti-inflammatory effects as a pre-treatment [76]. In relation to the action mechanism of GMP, at first, authors assumed that it was not related to anti-oxidative activity or to regulatory cell induction, as glutathione or transforming growth factor (TGF)-β levels in colon and Foxp-3 in ileum were not affected [76,77]. Later, trying to explain how GMP limits intestinal inflammation, they studied the action of GMP on monocyte cell line THP-1 and splenocytes, cells that produce cytokines with important roles in the inflammatory process. Curiously, GMP increased the secretion of TNF-α, IL-1β, and IL-8 by THP-1 cells (Figure 4D), and augmented the expression of Foxp-3, cyclooxygenase (COX)-2, and inducible oxide nitric synthase (iNOS) and the release of IL-10 and TNF-α by splenocytes (Figure 4A3) [68,79]. However, GMP inhibited IFN-γ and TNF-α secretion in ConA-stimulated splenocytes (Figure 4A1) [68]. Furthermore, when animals were orally administered with GMP, the ability to increase Foxp3 expression in spleen cells was retained, but secretion of cytokines by ex vivo ConA-stimulated splenocytes was not altered. Authors concluded that the intestinal anti-inflammatory action of GMP is probably mediated by the direct modulation of monocyte or splenocyte activity, especially by hampering the activation of T helper 1 (Th1) cells while favoring the differentiation of regulatory T (Treg) cells [68]. In the same way, in a mice model of famoxadone-induced colitis, orally GMP induced body weight restoration and intestinal injury reduction, in association with a decrease in CD4, CD8, mucosal addressin cell adhesion molecule-1 (MAdCAM), mitogen extracellular-signal-regulated kinase kinase-1 (MEKK1), and Smad7 (a molecule that transduces TGF-β signaling) intestinal expression [75]. Thus, GMP protection in colitis might be due to a decrease in T-lymphocyte gut infiltration, and to an upregulation of TGF-β and a downregulation of mitogen activation protein (MAP) kinase and NF-κB signaling pathways. Strikingly, orally administered GMP had an anti-inflammatory effect not as good as expected on two other experimental models of colitis in mice, the lymphocyte-transfer and DSS-induced models (Table 1) [80], pointing out that activity of GMP on intestinal inflammation depends on animal species and experimental model. All these experimental contributions culminated in a pilot study in patients with active ulcerative colitis that demonstrated that GMP used as a nutritional therapy complementary to standard treatment shows the same disease-modifying effect as mesalamine (standard treatment) dose escalation treatment, in addition to being safe and well accepted by patients [81].

In the last few years, our laboratory focused on the study of the immuno-modulatory activity of GMP in experimental models of allergies (revised in detail in Table 1). We found that oral administration of GMP before and during sensitization of rats with allergen induces a significant reduction in the level of allergen-specific IgE in serum, and decreases the proliferative response and the production of IL-13 by splenocytes in response to the allergen [70]. The treatment of animals with GMP also reduced the intensity of urticarial inflammatory reaction and protected animals from systemic anaphylaxis. Once we demonstrated the immuno-modulatory capacity of GMP on allergic sensitization and its beneficial effect on clinical signs associated with early-phase allergic reaction, we investigated whether GMP may impact on late-phase and chronic inflammatory reaction of allergy, choosing two experimental models that met these conditions, such as asthma and atopic dermatitis [85]. In both models, GMP was orally and prophylactically administered, that is to say, prior to and during pathology development. As expected, GMP intake resulted in a reduction of allergic immune response and of tissue inflammation and remodeling (Table 1) [83,84]. In both pathologies, expression of IL-5 and IL-13 was markedly inhibited in lung or skin, while that of IL-10 was increased. In experimental atopic dermatitis, we demonstrated the same protective effects when GMP was administered as post-treatment, with the exception of antipruritic action, although pre-treatment exerted a clearly superior effect in all analyzed variables. Our investigations then turned to the mechanism via which GMP modulates the allergic response. We analyzed levels of intestinal microbiota associated with protection in allergy, splenocyte production of regulatory cytokines in response to allergens, and number and function of mast cells (key cells in allergy). GMP administration increased the amount of intestinal *Lactobacillus* and *Bifidobacterium* of allergen-sensitized animals after three days, and that of *Bacteroides* after 17 days of GMP administration. Interestingly, ten days after GMP cessation, *Lactobacillus* and *Bacteroides* amounts were still elevated in animals gut. GMP intake also elevated the production of TGF-β by splenocytes of sensitized animals in response to allergen. In addition, GMP administration (and also in vitro incubation) impacted mast-cell function, inhibiting its activation and also the release of histamine in response to allergens, without altering mast-cell number in tissues (Figure 4E) [82].

In conclusion, GMP exerts important benefits on different inflammatory conditions, with the effect being greater when it is prophylactically used. Although more studies are needed, the action mechanism of GMP on immune response may be mediated by increasing healthy intestinal microbiota, inhibiting splenocyte proliferation, promoting a systemic and local regulatory environment, and also by directly modulating cell immune functions. However, we cannot exclude a possible effect of products derived from GMP digestion on in vivo immuno-modulatory activity.

## 4. Signaling Pathways Regulated or Activated by GMP or GMP-Hydrolyzed

At the present time, information about the mechanism that mediates GMP biological effects is scarce and is mostly generated from assays done with different cells of the immune system. Little is known about the signaling pathways modulated by GMP, and even less about the identity of the cellular receptors that are interacting with it.

### 4.1. Signaling Related with Immuno-Modulatory Activity

**Figure 5:**
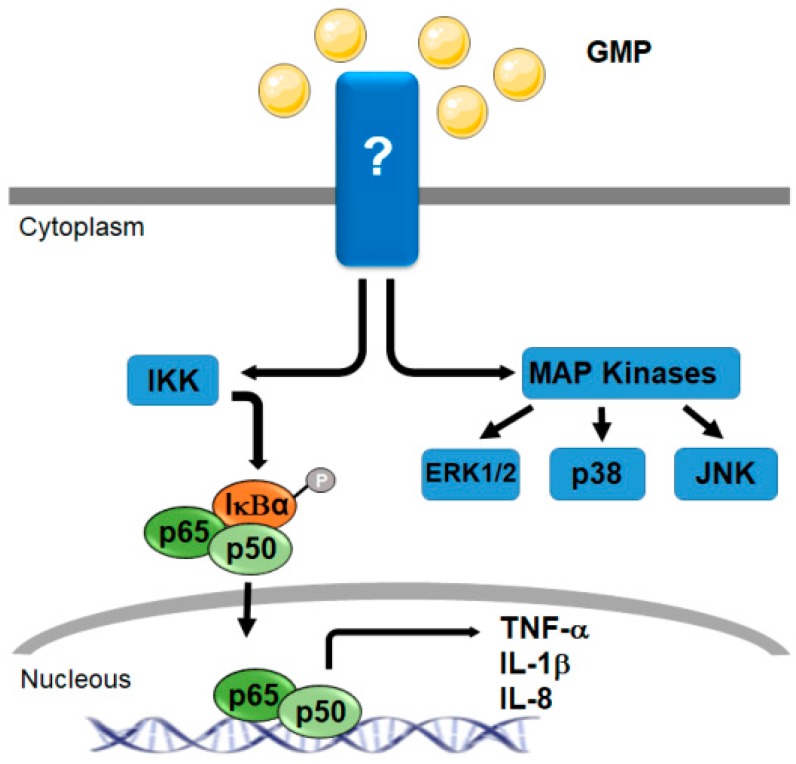
Signaling pathway involved in THP-1 human monocyte activation by glycomacropeptide. Through an unknown (?) receptor, glycomacropeptide (GMP) induces tumor necrosis factor (TNF)-α, interleukin (IL)-1β, and IL-8 production. Intracellular molecules that participate in the response of monocytes to GMP are mitogen-activated protein (MAP) kinases p38, c-Jun N-terminal kinase (JNK), and extracellular-signal-regulated protein kinases 1 and 2 (ERK1/2). The nuclear factor-κB (NF-κB) pathway is also involved as GMP activates the phosphorylation of inhibitor of κBα (IκBα) and the nuclear translocation of the p50 and p65 NF-κB subunits. IKK, kinase IκB; P, phosphate group.

As previously mentioned, it was demonstrated that GMP increases the expression of pro-inflammatory cytokines such as TNF-α, IL-1β, and IL-8 in the human monocyte cell line THP-1, in a dose-dependent way [79]. Using pharmacological inhibitors of some kinases involved in activation pathways in monocytes, authors analyzed intracellular molecules participating in signaling mediated by GMP. It was determined that the MAP kinases p38, c-Jun N-terminal kinase (JNK), and extracellular-signal-regulated protein kinase (ERK), and the NF-κB pathway were involved in the response of monocytes to GMP (Figure 5). In particular, it was determined that GMP activates the phosphorylation of inhibitor of κB (IkB)-α and the nuclear translocation of the NF-κB subunits p50 and p65. IL-8 secretion was not dependent on p38 activation. The results observed in the THP-1 cell line were also validated in human primary blood. This is the only work in which signaling pathways modulated by intact GMP were described and it suggests that GMP activates these cells of the immune system to boost the immune response [79].

As reviewed in a previous section, the immuno-modulatory activity of GMP was not only demonstrated by in vitro assays, but also in animal models. When GMP is administered orally to animals, the peptide is susceptible to be degraded by digestive enzymes, and it was reported that the effect of GMP on cells is different depending on whether it is intact or proteolyzed [71]. Thus, it is also important to know signaling pathways activated by the degradation products of GMP.

**Figure 6:**
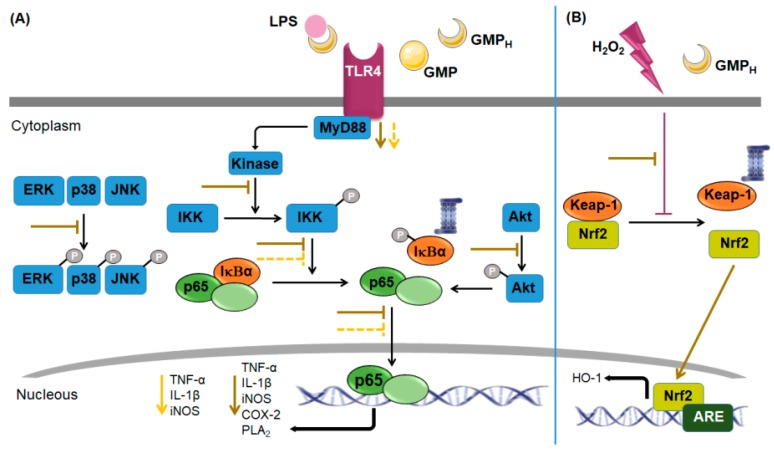
Signaling pathway involved in intact or papain-hydrolyzed glycomacropeptide modulation on RAW 264.7 macrophage response to lipopolysaccharide or hydrogen peroxide. (**A**) Glycomacropeptide (GMP) and a papain-hydrolyzed GMP (GMP_H_) downregulate pro-inflammatory response of RAW 264.7 macrophages induced by lipopolysaccharide (LPS). GMP_H_ is able to bind LPS and to suppress LPS binding to the macrophage surface. GMP_H_ diminishes the increase in myeloid differentiation primary response protein (MyD88) messenger RNA (mRNA) expression levels, inhibits phosphorylation and degradation of inhibitor of κBα (IκBα) and phosphorylation of kinase of IκB α/β (IKKα/β), and blocks p65 nuclear translocation in part by suppressing protein kinase B (Akt) phosphorylation. Additionally, it inhibits the phosphorylation of p-38, extracellular-signal-regulated protein kinase (ERK), and c-Jun N-terminal kinase (JNK) induced by LPS. GMP decreases MyD88 mRNA expression levels, p65 nuclear translocation, and phosphorylation and degradation of IκBα. Discontinuous lines indicate a minor effect. (**B**) GMP_H_ has antioxidant activity on hydrogen peroxide (H_2_O_2_)-stressed macrophages RAW 264.7. GMP_H_ blocks the inhibition of nuclear translocation of the nuclear factor erythroid 2-related factor 2 (Nrf2) induced by H_2_O_2_, and, as a consequence, increases heme oxygenase-1 (HO-1) expression. TLR4, Toll-like receptor 4; P, phosphate group; TNF-α, tumor necrosis factor-α; IL-1β, interleukin-1β; iNOS, inducible nitric oxide synthase; COX-2, cyclooxygenase-2; PLA2, phospholipase A2; Keap-1, kelch like E3 ubiquitin ligase complex-associated protein-1; ARE, antioxidant response element.

Cheng et al. [86] studied the effect of three enzymatic hydrolysates of GMP (papain, pepsin, and alcalase) on nitric oxide (NO) production by LPS-stimulated macrophages of the murine cell line RAW 264.7. Although an inhibitory effect was obtained with each hydrolysate, the best inhibitory activity was reported to papain-hydrolyzed GMP (GMP_H_); thus, the author decided to choose it for the rest of the study. It was demonstrated that GMP_H_ inhibits the inflammatory response and oxidative stress stimulated by LPS on macrophages of the murine cell line RAW 264.7 [86,87]. GMP_H_ significantly decreased NO and prostaglandin E2 production, intracellular reactive oxygen species (ROS) and malondialdehyde levels, and TNF-α, IL-1β, iNOS, COX-2, and phospholipase A_2_ messenger RNA (mRNA) expression. A minor inhibitory effect, although significant, on LPS-stimulated inflammatory response (TNF-α, IL-1β, iNOS) was observed also when cells were pre-incubated with intact GMP [86]. Results showed that GMP_H_ was able to bind directly to LPS and to suppress LPS binding to the macrophage surface [86]. Analyzing the pathways involved in LPS-induced Toll-like receptor 4 (TLR4) activation, GMP_H_ diminished the increase in TLR4 and MyD88 mRNA expression levels, blocked p65 nuclear translocation, and inhibited phosphorylation and degradation of IκBα and phosphorylation of kinase of IκB (IKK)α/β, stimulated by LPS in macrophages (Figure 6A) [86]. The inhibitory activity of GMP_H_ on LPS-induced NF-κB activity was mediated, at least in part, by suppressing protein kinase B (Akt) phosphorylation [84]. As authors used intact GMP as a control in their assays, results showed that it also decreased MyD88 mRNA expression levels, p65 nuclear translocation, and phosphorylation and degradation of IκBα, although the effects were not as great as with its hydrolyzed counterpart [86]. Moreover, GMP_H_ inhibited the increase in phosphorylated (p)-p38/p-38, p-ERK/ERK and p-JNK/JNK ratio induced by LPS [87]. All these results suggest that the anti-inflammatory effect mediated by GMP_H_ in LPS-stimulated macrophages is mediated by the negative regulation of the MyD88/NF-κB and MAP kinase pathways.

In other interesting work, the same researchers also determined that intact GMP and its papain-hydrolyzed counterpart have antioxidant activity on macrophages (RAW 264.7), when cells were stressed by hydrogen peroxide (H_2_O_2_) [88]. Antioxidant and cytoprotective effects of GMP_H_ were significantly higher than that of intact peptide. Authors demonstrated that the hydrolyzed peptide increases the nuclear translocation of the nuclear factor erythroid 2-related factor 2 (Nrf2), as well as heme oxygenase-1 (HO-1) expression at mRNA and protein level, at least in part, via the intracellular signaling mediated by ROS (Figure 6B) [88].

### 4.2. Signaling Associated with Metabolism Modulating Activity

**Figure 7:**
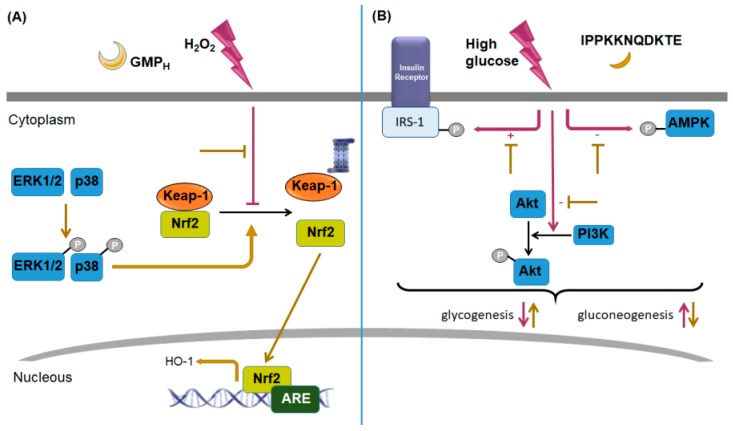
Signaling pathway involved in papain-hydrolyzed glycomacropeptide modulation on HepG2 cell response to hydrogen peroxide or to high-glucose conditions. (**A**) A papain-hydrolyzed glycomacropeptide (GMP_H_) shows hepatoprotection and antioxidant activity on hydrogen peroxide (H_2_O_2_)-induced oxidative damaged in hepatocytes HepG2. Via extracellular-signal-regulated protein kinase (ERK1/2) and mitogen-activated protein (MAP) kinase p38 activation (phosphorylation), GMP_H_ blocks the inhibition of nuclear translocation of the nuclear factor erythroid 2-related factor 2 (Nrf2) induced by H_2_O_2_, and, as a consequence, increases heme oxygenase-1 (HO-1) expression. (**B**) The peptide IPPKKNQDKTE derived from GMP_H_ improves impaired glucose metabolism in high-glucose-induced insulin-resistant HepG2 cells, by increasing glucose intake, promoting glycogenesis, and inhibiting gluconeogenesis. IPPKKNQDKTE blocks the increase in insulin receptor substrate-1 (IRS-1) phosphorylation, and the decrease in adenosine monophosphate (AMP)-activated protein kinase (AMPK) phosphorylation and phosphatidylinositol 3-kinase (PI3K)/protein kinase B (Akt) activation, induced by high-glucose conditions. P, phosphate group; Keap-1, kelch like E3 ubiquitin ligase complex-associated protein-1; ARE, antioxidant response element.

Oxidative stress may lead to damage to cellular macromolecules and it is implicated in the development of several metabolic diseases, including type-2 diabetes, obesity, and non-alcoholic fatty liver disease. It was demonstrated that GMP_H_ generates cytoprotection on H_2_O_2_-induced damage in hepatocytes HepG2. Authors showed that MAP kinases p38 and ERK1/2, but not JNK, participate in Nrf2-medited HO-1 expression associated with hepatoprotection (Figure 7A) [89]. It could be possible that these two MAP kinases also participate in the aforementioned cytoprotective effect of GMP_H_ on macrophages (RAW 264.7).

Recently, using HPLC separation and analysis by liquid chromatography coupled with electrospray ionization tandem mass spectrometry, Song et al. [90] identify a peptide from GMP_H_, which corresponds to the amino-acid sequence IPPKKNQDKTE. In insulin-resistant HepG2 cells induced by high glucose, this peptide prevented the decrease in cellular glucose uptake and the increase in gluconeogenic enzymes, as well as generated an increase in intracellular glycogen by regulating the phosphorylation levels of key glycogenic proteins. Authors showed that the effect was mediated by the activation of the phosphatidylinositol 3-kinase (PI3K)/Akt signaling pathway, associated with decreased phosphorylation of insulin receptor substrate-1 (IRS-1) and increased phosphorylation of AMP-activated protein kinase (AMPK) (which were respectively increased and decreased in high-glucose conditions) (Figure 7B) [90]. It is important to mention that the peptide IPPKKNQDKTE does not present sites of post-translational modifications such as phosphorylations or glycosylations; thus, its effect in insulin-resistant HepG2 cells is exclusively dependent on its amino-acid sequence.

In summary, most of the information about GMP in the context of signaling pathways was obtained from studies using GMP_H_. As papain is not present in the digestive system, studies focusing on analyzing intracellular pathways modulated by pepsin-, trypsin-, or chymotrypsin-hydrolyzed GMP may generate more accurate information about how orally ingested GMP mediates its biological effects.

## 5. Conclusions

GMP is a milk-derived bioactive peptide with potential health-enhancing benefits. Using in vitro assays, interesting biological effects of GMP were described, highlighting that the structure of the peptide as a major determinant in its biological functions. However, more work is needed on strengthening the knowledge about the bioactivity of orally administered GMP. Once ingested, it is susceptible to be enzymatically hydrolyzed during digestion, although it can also be absorbed intact and distributed by blood vessels, or it can reach the large intestine to be fermented by microbiota. New studies that analyze the biological effect of GMP-hydrolysates generated by digestive enzymes are of paramount importance to establish a cause–effect relationship with more accuracy. To date, animal models yielded promising results about GMP in the promotion of health and in the prevention of some inflammatory diseases, such as metabolic diseases, colitis, or allergy. An in-depth exploration of GMP action mechanism at the physiological, cellular, and molecular levels will speed up the development of clinical studies with GMP in human health and its incorporation as an ingredient in functional foods, dietary supplements, and nutraceuticals. In addition, deciphering the structural features that confer its bioactivity, the mechanism via which GMP exerts its activity, and the signaling molecules involved will allow inferring and analyzing other possible activities and uses of GMP in health.

## Figures and Tables

**Table 1:** Glycomacropeptide in rodent models of inflammatory diseases.

Model		Specie and Strain	GMP Doses and Treatment	Effects and No Effects, Relative to Comparison Groups	References
Colitis	TNBS- induced	Female Wistar rats	500 mg/kg/day; Pre- and post-treatment	**Effects:** ↓ Colonic weight:length ratio ↓ Necrosis extension ↓ Damage score ↑ Food intake ↓ Weight loss ↓ Colonic AP activity ↓ iNOS expression in colon ↓ mRNA levels of IL-1β, IL-1ra, MUC4 and TFF3 in colon Intensity of effects: pre-treatment > post-treatment. **No effects on:** - Glutathione reduced levels in colon - Colonic mRNA expression of TGF-β	[76]
	DSS-induced	Female Wistar rats	500 mg/kg/day; Pre-treatment	**Effects:** ↓ Damage score ↑ Food intake ↓ ConA-stimulated IFN-γ secretion on mesenteric node cells ↓ mRNA levels of IL-1β, IL-6, IL-23, IL-17, IL-10, TGF- β, and Foxp3 in colon **No effects on:** - Lost of body weight - Increased AP and MPO colonic activity - Augmented IL-2 secretion stimulated by ConA on mesenteric node cells	[78]
	Lymphocyte transfer model	Female C57BL/6 mice	15 mg/day; Post-treatment	**No effects on:** - Lost of body weight - Damage score - Increased colonic weight:length ratio - Augmented AP and MPO colonic activity - Increased mRNA levels of REG3γ, S100A8, CXCL1, and IL-1β in colon - Increased TNF-α, IL-17, IL-10, and IL-6 secretion stimulated by ConA on mesenteric node cells	[80]
	DSS-induced	Female C57BL/6 mice	15 mg/day; Pre-treatment	**Effects:** ↑ Up-regulated IL-10 secretion by ConA-stimulated mesenteric node cells **No effects on:** - Lost of body weight - Damage score - Increased colonic weight–length ratio and colon length - Augmented AP and MPO colonic activity - Increased IL-17 secretion stimulated by ConA on mesenteric node cells	[80]
	OXZ-induced	Male BALB/c mice	50 mg/kg/day; Treatment	**Effects:** ↓ Weight loss ↓ Intestinal morphological injury score ↓ Increased expression of CD4, CD8, and MAdCAM-1 in lamina propria, and MEKK1 and Smad7 in intestinal tissue ↑ Decreased levels of IgAs in lamina propria **No effects on:** - Smad3 levels in intestinal tissue	[75]
Ileitis	TNBS-induced	Female Wistar rats	500 mg/kg/day; Pre-treatment	**Effects:** ↓ Intestinal necrosis extension ↓ Damage score ↓ AP and MPO intestinal activity ↓ iNOS expression in ileum ↓ IL-1β, TNF-α, IL-1ra, IL-17, and TFF3 mRNA levels in ileal tissue **No effects on:** - Decreased weight loss or increased food intake - Increased COX-2 levels - Increased secretion of TNF-α, IFN-β, and IL-2 by in vitro stimulated mesenteric lymph node cells - Increased levels of RNA to Foxp3 in ileum	[77]
Hives and anafilaxis	OVA-induced	Male Wistar rats	500 mg/kg/day; Pre-treatment	**Effects:** ↓ Allergen-specific IgE ↓ Splenocyte proliferation induced by allergen and ConA ↓ IL-13 and ↑ TGF-β splenocyte secretion stimulated by allergen ↑ Intestinal *Lactobacillus*, *Bifidobacterium*, and *Bacteroides* ↓ Intensity of inflammatory reaction and cutaneous mast-cell activation induced by i.d. allergen injection ↓ Score of anaphylactic shock and ↑ rectal temperature and rate of survival, induced by i.v. allergen injection **No effects on:** - Increased secretion of IL-10 by splenocytes in response to allergen - Number of cutaneous mast cells	[70,82]
Asthma	OVA-induced	Male Wistar rats	500 mg/kg/day; Pre-treatment	**Effects:** ↓ Allergen-specific IgE ↓ Eosinophils and ↑ lymphocytes in blood ↓ Total cells in BALF, particularly basophils, neutrophils and lymphocytes ↓ Lung eosinophil infiltration, goblet cell hyperplasia and collagen deposition ↓ Lung TGF-β expression ↓ IL-5 and IL-13, but ↑ IL-10 mRNA levels in lung **No effects on:** - Increased eosinophils number in BALF	[83]
Atopic dermatitis	DNCB-induced	Male Wistar rats	500 mg/kg/day; Pre- and post-treatment	**Effects:** ↓ Total IgE ↓ Cutaneous inflammatory process induced by topical application of allergen ↓ Pruritus ↓ Skin eosinophil infiltration and mast-cell hyperplasia ↓ IL-4, IL-5, and IL-13, but ↑ IL-10 mRNA levels in skin. Intensity of effects: pre-treatment > post-treatment. **No effects on:** - Pruritus when GMP was administered as post-treatment	[84]

**Abbreviations:** GMP, glycomacropeptide; TNBS, trinitrobenzenesulfonic acid; AP, alkaline phosphatase; iNOS, inducible oxide nitric synthase; mRNA, messenger RNA IL, interleukin 1β; IL-1ra, interleukin 1 receptor antagonist; MUC4, mucin 4; TFF3, trefoil factor 3; DSS, dextran sulfate sodium; ConA, concanavalin A; IFN, interferon; TGF, transforming growth factor; Foxp-3, forkhead box protein; MPO, myeloperoxidase; REG3γ, islet-derived protein 3γ; s100A8, S100 calcium binding protein A8; CXCL, CXC chemokine ligand; TNF, tumoral necrosis factor; OXZ, famoxadone; CD, cluster of differentiation; MAdCAM-1, mucosal addressin cell adhesion molecule-1; MEKK1, mitogen/extracellular-signal-regulated kinase kinase-1; Smad, signaling molecules that transduce TGF-β signaling; Ig, immunoglobulin; COX-2, cyclooxygenase 2; OVA, ovalbumin; i.d., intradermal; i.v., intravenous; BALF, bronchoalveolar lavage fluid; DNCB; 2,4-dinitrochlorobenzene; ↓, decreased; ↑, increased. Pre-treatment: from days before and during pathology induction to the end of the model; Treatment: from the first day of pathology induction to the end of the model; Post-treatment: once pathology is established and damage is induced to the end of the model.
